# Serum macrophage migration inhibitory factor as a predictor of radiographic progression in ankylosing spondylitis: a retrospective cohort study

**DOI:** 10.1007/s10067-026-08063-5

**Published:** 2026-04-16

**Authors:** Yasser A. Elmotaleb Gazar, Sherif Ismail, Saad Ghanem, Mohamed Magdy Ghit, Adel Ibrahim Azzam

**Affiliations:** 1 Rheumatology and Rehabilitation Department, Faculty of Medicine, Al-Azhar University, Cairo, Egypt; 2 Rheumatology and Rehabilitation, Internal Medicine Department, Medical Research and Clinical Studies Institute, National Research Center, Giza, Egypt; 3 Rheumatology and Rehabilitation Department, Faculty of Medicine, Al-Azhar University, Damietta, Egypt

**Keywords:** Ankylosing spondylitis, Biomarker, Macrophage migration inhibitory factor, MIF, Modified Stoke Ankylosing Spondylitis Spine Score, MSASSS, Radiographic progression

## Abstract

**Objective:**

Macrophage migration inhibitory factor (MIF) is a pleiotropic pro-inflammatory cytokine implicated in the pathogenesis of ankylosing spondylitis (AS). This study aimed to evaluate whether serum MIF levels measured at baseline are associated with radiographic progression in AS patients assessed retrospectively over time.

**Methods:**

This retrospective cohort study with concurrent healthy controls included 50 AS patients meeting the modified New York criteria and 60 age- and sex-matched healthy controls. Serum MIF was measured by ELISA at baseline. Clinical assessment included Bath Ankylosing Spondylitis Disease Activity Index (BASDAI), erythrocyte sedimentation rate (ESR), and C-reactive protein (CRP). Radiographic progression was assessed retrospectively using the modified Stoke Ankylosing Spondylitis Spine Score (mSASSS) on radiographs obtained at baseline and at least 12 months later (median interval 24 months, range 12–36). Progressors were defined as patients with annualized mSASSS increase ≥ 1 unit/year. Two independent readers scored all radiographs while blinded to clinical data and time sequence.

**Results:**

Serum MIF levels were significantly elevated in AS patients compared to controls (median 43.0 ng/mL vs. 11.2 ng/mL, p < 0.0001). Among AS patients, 15 (30%) were classified as progressors. After adjusting for age, sex, disease duration, baseline mSASSS, smoking, and ESR in multivariable logistic regression, elevated MIF remained independently associated with progression (OR 1.85 per 10 ng/mL increase, 95% CI 1.08–3.18, p = 0.028). MIF levels correlated with disease duration (ρ = 0.299, *p *= 0.035), ESR (ρ = 0.669, *p* < 0.0001), and mSASSS score (ρ = 0.324, *p* = 0.021). ROC analysis demonstrated that MIF > 51 ng/mL discriminated progressors from non-progressors with AUC 0.746 (95% CI 0.599–0.893), sensitivity 53.3%, specificity 94.3%, positive predictive value 80.0%, and negative predictive value 82.5%.

**Conclusion:**

Baseline serum MIF is independently associated with subsequent radiographic progression in AS patients after adjustment for key confounders. While sensitivity is modest (53%), the high specificity (94%) suggests that elevated MIF (> 51 ng/mL) may help identify patients at higher risk for progressive spinal damage.

**Table:** 

**Key Points** • *Baseline serum MIF levels are independently associated with subsequent radiographic progression in AS after adjusting for age, sex, disease duration, baseline damage, smoking, and ESR.* • *MIF >51 ng/mL predicts progression with high specificity (94%) but modest sensitivity (53%), suggesting utility for identifying high-risk patients rather than screening all patients.* • *MIF correlates moderately with ESR (ρ=0.669) but shows independent prognostic value in multivariable models, consistent with MIF's dual role in driving inflammation and bone formation.* • *Rigorous radiographic methodology (blinded dual readers, ICC 0.94) and detailed ELISA protocols strengthen confidence in findings, though prospective validation in larger cohorts is needed.*

## Introduction

Ankylosing spondylitis (AS) is a chronic inflammatory disease predominantly affecting the axial skeleton, characterized by inflammation of the sacroiliac joints and spine, leading to pain, stiffness, and progressive structural damage [1, 2]. As the prototype of spondyloarthritides, AS affects approximately 0.5–1% of the population globally, with significant variability in disease progression [3]. Approximately 30–40% of patients experience rapid radiographic progression marked by osteoproliferation and spinal fusion, resulting in substantial functional impairment [4].

Despite strong HLA-B27 association (present in 90% of AS patients), the pathogenesis linking chronic inflammation to paradoxical new bone formation remains incompletely understood [5]. Current biomarkers, including acute-phase reactants such as C-reactive protein (CRP) and erythrocyte sedimentation rate (ESR), inadequately predict which patients will develop severe structural damage [6]. This represents an important unmet clinical need, as early identification of high-risk patients could inform treatment intensification decisions.

Macrophage migration inhibitory factor (MIF) is a pleiotropic pro-inflammatory cytokine that serves as an upstream regulator of innate immunity [7]. Unlike typical cytokines, MIF is constitutively expressed and stored intracellularly, allowing rapid release upon inflammatory stimuli. MIF overrides glucocorticoid-mediated immunosuppression and sustains production of pro-inflammatory mediators including TNF-α, IL-1β, IL-6, and IL-17 [8]. Emerging evidence suggests MIF may possess unique properties linking inflammation to bone formation in AS [9].

Elevated serum MIF levels have been reported in AS patients, with correlation to disease activity markers [10]. Importantly, Ranganathan et al. demonstrated that MIF not only drives inflammation but also directly promotes osteoblast differentiation and mineralization through Wnt/β-catenin pathway activation [9]. Furthermore, MIF levels predicted radiographic progression over time in their prospective cohort. These findings suggest MIF may serve as both a pathogenic mediator and prognostic biomarker in AS.

However, the relationship between MIF and radiographic progression requires validation in independent cohorts, particularly with rigorous adjustment for potential confounders such as age, disease duration, and critically, baseline radiographic damage, which is a strong predictor of future progression. Furthermore, the optimal MIF threshold for clinical decision-making remains undefined. This study aimed to: (1) compare serum MIF levels between AS patients and healthy controls, (2) evaluate whether baseline MIF levels are independently associated with subsequent radiographic progression after adjusting for key confounders, (3) assess the discriminative performance of MIF for identifying progressors, and (4) determine a clinically applicable MIF threshold.

## Patients and methods

### Study design

This was a retrospective cohort study of AS patients with concurrent healthy control comparison for baseline MIF levels. AS patients were identified from rheumatology clinics at EL-Hussein University Hospital, Cairo, Egypt, between January 2017 and December 2019. The primary outcome was radiographic progression assessed retrospectively using serial radiographs. The study was approved by the institutional ethics committee of Al-Azhar University Faculty of Medicine (Registration number 00000030) and conducted in accordance with the Declaration of Helsinki. All participants provided written informed consent.

**Study timeline:** Baseline serum samples were collected between January-June 2017. Baseline radiographs were obtained within 3 months of blood sampling (median interval 1.5 months, range 0–3). Follow-up radiographs were retrieved retrospectively from medical records, with median interval of 24 months (range 12–36 months, IQR 18–30 months) between baseline and follow-up imaging.

### Study participants

**AS Patients:** Fifty patients with AS meeting the modified 1984 New York criteria [11] were enrolled. Inclusion criteria: (1) age 18–60 years, (2) disease duration ≥ 2 years, (3) availability of radiographs at baseline and at least 12 months later for mSASSS scoring. Exclusion criteria: (1) other spondyloarthropathies (psoriatic arthritis, enteropathic arthritis, reactive arthritis), (2) rheumatoid arthritis or other inflammatory arthritis, (3) other autoimmune diseases, (4) diabetes mellitus (to avoid potential confounding effects on MIF levels [12]), (5) active infection or malignancy, (6) corticosteroid use within 3 months of enrollment (as MIF is regulated by glucocorticoids).

**Healthy Controls:** Sixty age- and sex-matched healthy volunteers were recruited from hospital staff and community volunteers. Controls were screened clinically to exclude: (1) any rheumatic or autoimmune disease, (2) chronic inflammatory conditions, (3) diabetes mellitus, (4) active infection or other overt inflammatory conditions, (5) use of corticosteroids, NSAIDs, or immunomodulatory medications within 3 months, and (6) first-degree relatives with spondyloarthritis. Controls underwent identical blood sampling protocols as patients but did not undergo radiographic evaluation. CRP was measured as part of baseline laboratory characterization and was not used as a strict standalone inclusion cutoff.

### Clinical assessment

All AS patients underwent comprehensive clinical evaluation at baseline, including detailed medical history (age, sex, smoking status [current smoker vs. never/former], disease duration from first symptoms, comorbidities, and current medications including NSAIDs, conventional synthetic disease-modifying anti-rheumatic drugs [csDMARDs primarily sulfasalazine and methotrexate], and TNF inhibitors). Disease activity was assessed using the validated Bath Ankylosing Spondylitis Disease Activity Index (BASDAI) [13], a patient-reported outcome measure consisting of six 10-cm visual analog scales addressing fatigue, spinal pain, peripheral joint pain, enthesitis, morning stiffness severity, and morning stiffness duration (total score 0–10, with higher scores indicating greater disease activity).

### Laboratory investigations

Blood samples were collected after overnight fasting between 8:00–10:00 AM to minimize diurnal variation. Serum was separated within 2 h of collection by centrifugation at 3000 g for 15 min at 4 °C, aliquoted into polypropylene cryovials, and stored at −80°C until batch analysis. All samples underwent a maximum of one freeze–thaw cycle prior to MIF measurement.

**Standard laboratory parameters:** Complete blood count was performed using automated hematology analyzer (Sysmex XN-1000, Sysmex Corporation, Kobe, Japan). ESR was measured by Westergren method (normal < 20 mm/h for males, < 30 mm/h for females). CRP was quantified by high-sensitivity immunoturbidimetric assay (Roche Cobas c501, Roche Diagnostics, Basel, Switzerland) with lower detection limit 0.15 mg/L and upper limit of normal 5 mg/L. Lipid profile and HbA1c were measured using standard enzymatic methods. HLA-B27 status was determined by flow cytometry.

**MIF measurement:** Serum MIF concentrations were measured in duplicate using a commercially available enzyme-linked immunosorbent assay (Human MIF Quantikine ELISA Kit, Catalog #DMF00B, R&D Systems, Minneapolis, MN, USA) according to manufacturer's instructions. The assay has a detection range of 0.031–2.0 ng/mL. Because preliminary testing indicated that undiluted AS patient sera exceeded this range, samples were initially diluted 1:25 in calibrator diluent prior to assay. Samples that remained above the upper calibration limit after 1:25 dilution were re-assayed at higher dilution factors as needed, and final MIF concentrations reported throughout this manuscript are back-calculated values based on the applied dilution factor, expressed in ng/mL. The assay demonstrates intra-assay coefficient of variation (CV) < 5% and inter-assay CV < 8%. Laboratory personnel performing MIF assays were blinded to patient clinical data, radiographic findings, and progression status. All samples were analyzed in a single batch to eliminate inter-assay variability. The mean of duplicate measurements was used for analysis; samples with CV > 10% between duplicates (*n* = 2) were re-analyzed.

### Radiographic assessment

Anteroposterior and lateral radiographs of the cervical spine and anteroposterior radiographs of the lumbar spine were obtained using standardized positioning protocols at both baseline and follow-up. Radiographic damage was quantified using the modified Stoke Ankylosing Spondylitis Spine Score (mSASSS) [14], which assesses the anterior vertebral corners of the cervical spine (lower border of C2 to upper border of T1) and lumbar spine (lower border of T12 to upper border of S1), totaling 24 sites (12 per region). Each site was scored as: 0 = normal, 1 = erosion, sclerosis, or squaring, 2 = syndesmophyte, 3 = total bony bridge (maximum possible score: 72).

**Reader methodology:** All radiographs were scored independently by two trained readers (SI and YAEG, both rheumatologists with > 5 years experience in mSASSS scoring) who were blinded to: (1) patient clinical data including MIF levels and other laboratory results, (2) temporal sequence of radiographs (baseline vs. follow-up), and (3) each other's scores. Prior to study scoring, both readers completed a calibration exercise using 10 radiograph sets not included in the study, achieving inter-reader agreement with intraclass correlation coefficient (ICC) > 0.90. Discrepancies of ≥ 2 points in total mSASSS between readers were adjudicated by joint review with a third senior reader (AIA), with consensus reached for final score. For the study cohort, inter-reader reliability was ICC = 0.94 (95% CI 0.91–0.96) for total mSASSS and ICC = 0.91 (95% CI 0.87–0.94) for change scores. The mean of the two readers' scores was used for analysis except when adjudication occurred, in which case the consensus score was used.

**Progression definition:** For each patient, the annualized change in mSASSS was calculated as: (follow-up mSASSS—baseline mSASSS)/(interval in years). Radiographic progressors were defined as patients with annualized mSASSS increase ≥ 1 unit per year, consistent with definitions used in prior AS progression studies [9, 15]. This threshold is clinically meaningful as it exceeds the smallest detectable change for mSASSS (approximately 0.5–1.0 units) [16]. Patients with annualized change < 1 unit per year were classified as non-progressors. The 1-unit/year threshold was pre-specified prior to analysis.

### Statistical analysis

Sample size was determined pragmatically based on available AS patients with serial radiographs during the study period. Post-hoc power calculation indicated 80% power to detect a moderate effect size (Cohen's d = 0.75) difference in MIF between progressors and non-progressors with α = 0.05, given the observed 15:35 ratio.

Continuous variables were assessed for normality using Shapiro–Wilk test and visual inspection of Q-Q plots. Due to non-normal distributions, continuous variables are presented as median with interquartile range (IQR), and group comparisons used Mann–Whitney U test. Categorical variables are presented as frequencies and percentages, with group comparisons using Chi-square or Fisher's exact test (when expected cell counts < 5). For comparison of MIF levels across groups, effect sizes are reported as median differences with 95% confidence intervals estimated by bootstrap methods (10,000 replications).

Associations between MIF and continuous clinical/laboratory parameters were assessed using Spearman's rank correlation coefficient (ρ). The strength of correlation was interpreted as: |ρ|< 0.3 weak, 0.3–0.7 moderate, > 0.7 strong.

**Multivariable modeling:** To assess the independent association between baseline MIF and radiographic progression while adjusting for potential confounders, we constructed a multivariable logistic regression model with progression status (yes/no) as the dependent variable. Predictor variables were selected a priori based on clinical knowledge and prior literature, not through stepwise selection. The model included: (1) age (years, continuous), (2) sex (male vs. female), (3) disease duration (years, continuous), (4) baseline mSASSS (continuous, to account for baseline damage level), (5) current smoking status (yes/no), (6) ESR (mm/h, continuous, as representative inflammatory marker), and (7) MIF (ng/mL, continuous). Given the small number of outcome events (*n *= 15 progressors), we limited the model to 7 predictors, maintaining approximately 2 events per variable—below the traditional guideline of 10 events per variable but adequate for exploratory analyses with cautious interpretation [17]. Results are presented as odds ratios (OR) with 95% confidence intervals. For clinical interpretability, ORs for continuous variables are scaled to clinically meaningful units: MIF per 10 ng/mL increase, ESR per 10 mm/h increase, age per 5-year increase, disease duration per 5-year increase, and baseline mSASSS per 1-unit increase. We also performed sensitivity analyses using Firth's penalized likelihood logistic regression to address potential small-sample bias.

Model diagnostics included assessment of multicollinearity (variance inflation factors, VIF < 5 considered acceptable), influential observations (Cook's distance), and model fit (Hosmer–Lemeshow goodness-of-fit test).

**Discriminative performance:** Receiver operating characteristic (ROC) curve analysis evaluated MIF's ability to discriminate progressors from non-progressors. The area under the ROC curve (AUC) was calculated with 95% confidence intervals estimated by DeLong method. The optimal cut-off was determined using Youden's index (maximizing sensitivity + specificity—1). At this cut-off, we calculated sensitivity, specificity, positive predictive value (PPV), and negative predictive value (NPV) with 95% confidence intervals estimated by exact binomial methods. PPV and NPV were calculated at the observed 30% prevalence of progressors in this cohort. To assess incremental value of MIF beyond conventional markers, we compared AUC of models with and without MIF using DeLong test for comparing correlated ROC curves.

**Missing data:** Missing data were minimal (< 2% for any variable). HLA-B27 status was available for all 50 AS patients. One control had missing lipid profile data. All other variables had complete data. Given the minimal missingness, we used complete case analysis without imputation.

All statistical tests were two-sided with significance level α = 0.05. Analyses were performed using IBM SPSS Statistics Version 20 (IBM Corp., Armonk, NY) and R version 4.1.0 (R Foundation for Statistical Computing, Vienna, Austria). This study follows the STROBE (Strengthening the Reporting of Observational Studies in Epidemiology) guidelines for reporting of observational studies.

## Results

### Study population characteristics

During the study period, 67 AS patients were screened; 17 were excluded (8 had disease duration < 2 years, 6 lacked adequate follow-up radiographs, 2 had diabetes mellitus, 1 had concurrent rheumatoid arthritis). The final cohort included 50 AS patients (33 male, 66%) with median age 38 years (IQR 33–43) and 60 healthy controls (40 male, 67%) with median age 37 years (IQR 32–42). Groups were well-matched for age (*p* = 0.68) and sex (*p *= 0.91).

Among AS patients, median disease duration from symptom onset was 15 years (IQR 11–20), median BASDAI score was 3.5 (IQR 2–4), and 64% were HLA-B27 positive. The median interval between baseline and follow-up radiographs was 24 months (IQR 18–30, range 12–36 months). Most patients had predominantly axial disease (78%), with 22% having peripheral joint involvement. Current medications included NSAIDs in 54%, csDMARDs (primarily sulfasalazine or methotrexate) in 88%, and TNF inhibitors in 14% of patients. Complete demographic and clinical characteristics are presented in Table 1.

**Table 1:** Baseline characteristics: AS patients versus healthy controls

Characteristic	AS patients (*n* = 50)	Healthy controls (*n* = 60)	*P* value
Age (years)	38 (33–43)	37 (32–42)	0.68
Male sex, *n* (%)	33 (66)	40 (67)	0.91
BMI (kg/m^2^)	26.2 (23.5–28.9)	25.8 (23.1–28.3)	0.62
Current smoking, *n* (%)	20 (40)	18 (30)	0.26
Hypertension, *n* (%)	9 (18)	8 (13)	0.49
Disease duration (years)	15 (11–20)	—	—
HLA-B27 positive, *n* (%)	32 (64)	—	—
BASDAI score	3.5 (2–4)	—	—
Hemoglobin (g/dL)	12.4 (11.2–13.1)	12.9 (11.5–13.6)	0.14
Platelets (× 10^3^/µL)	204 (167–221)	188 (168–207)	0.26
Total leukocytes (× 10^3^/µL)	5.4 (4.3–7.4)	5.9 (4.5–8.4)	0.51
ESR (mm/h)	30.6 (25.4–40.5)	9.6 (5.5–14.0)	< 0.0001
CRP (mg/L)	14.9 (8.1–22.4)	6.9 (2.8–11.6)	0.0016
Total cholesterol (mg/dL)	179.9 (167–195.6)	174.1 (165.4–190.7)	0.51
Triglycerides (mg/dL)	166.5 (149–179.2)	136 (121.5–187.2)	0.14
HbA1c (%)	3.5 (3.1–4.3)	4.0 (3.4–4.2)	0.37

Values are median (IQR) for continuous variables or *n* (%) for categorical variables. *BMI* Body mass index, *BASDAI* Bath Ankylosing Spondylitis Disease Activity Index, *ESR* Erythrocyte sedimentation rate, *CRP* C-reactive protein, *IQR* Interquartile range

### Serum MIF levels: as patients versus healthy controls

Serum MIF levels were significantly elevated in AS patients compared to healthy controls [median 43.0 ng/mL (IQR 37.5–48.3) vs. 11.2 ng/mL (IQR 8.6–13.1), *p* < 0.0001], representing a median difference of 31.8 ng/mL (95% CI 29.1–34.6). The effect size was very large (Cohen's d = 3.21). Similarly, ESR was significantly higher in AS patients [median 30.6 mm/h (IQR 25.4–40.5) vs. 9.6 mm/h (IQR 5.5–14.0), p < 0.0001], and CRP was elevated [median 14.9 mg/L (IQR 8.1–22.4) vs. 6.9 mg/L (IQR 2.8–11.6), *p* = 0.0016]. No significant differences were observed between groups in hemoglobin, platelets, total leukocyte count, total cholesterol, triglycerides, or HbA1c (Table 2).

**Table 2:** Serum MIF and inflammatory markers

Variable	AS patients (*n* = 50)	Controls (*n* = 60)	Median diff (95% CI)	*P* value
MIF (ng/mL)	43.0 (37.5–48.3)	11.2 (8.6–13.1)	31.8 (29.1–34.6)	< 0.0001
ESR (mm/h)	30.6 (25.4–40.5)	9.6 (5.5–14.0)	21.0 (16.2–25.8)	< 0.0001
CRP (mg/L)	14.9 (8.1–22.4)	6.9 (2.8–11.6)	8.0 (3.4–12.6)	0.0016

Values are median (IQR). *MIF* Macrophage migration inhibitory factor, *ESR* Erythrocyte sedimentation rate, *CRP* C-reactive protein, *CI* Confidence interval

### Radiographic progression: progressors versus non-progressors

Among the 50 AS patients, 15 (30%) were classified as radiographic progressors (annualized mSASSS change ≥ 1 unit/year) and 35 (70%) as non-progressors. The median annualized mSASSS change was 2.1 units/year (IQR 1.3–3.2) in progressors versus 0.3 units/year (IQR 0–0.6) in non-progressors (*p* < 0.0001). The median radiograph interval did not differ between groups: 24 months (IQR 18–30) for progressors vs. 24 months (IQR 18–28) for non-progressors (*p* = 0.81).

### Baseline characteristics by progression status

Progressors were significantly older than non-progressors [median 42 years (IQR 37–47) vs. 37 years (IQR 33–39), *p *= 0.023], more likely to be male (87% vs. 54%, *p *= 0.03), had longer disease duration [median 20 years (IQR 17–23) vs. 13 years (IQR 11–18), *p* = 0.0028], higher baseline mSASSS [median 16 (IQR 12–22) vs. 8 (IQR 4–11), *p* < 0.0001], and higher prevalence of current smoking (67% vs. 29%, *p* = 0.012). There were no significant differences in BASDAI score, hypertension, medication use (NSAIDs, csDMARDs, TNF inhibitors), or HLA-B27 positivity between groups (Table 3).

**Table 3:** Baseline characteristics by progression status

Characteristic	Non-progressors (*n* = 35)	Progressors (*n* = 15)	*P* value
Age (years)	37 (33–39)	42 (37–47)	0.023
Male sex, *n* (%)	19 (54)	13 (87)	0.03
Disease duration (years)	13 (11–18)	20 (17–23)	0.0028
Current smoking, *n *(%)	10 (29)	10 (67)	0.012
Hypertension, *n* (%)	6 (17)	3 (20)	0.81
BASDAI score	4 (2–4)	3 (2–4)	0.93
HLA-B27 positive, *n* (%)	20 (57)	12 (80)	0.13
NSAIDs use, *n *(%)	18 (51)	9 (60)	0.58
csDMARDs use, *n *(%)	30 (86)	14 (93)	0.45
TNF inhibitor use, *n *(%)	5 (14)	2 (13)	0.93
MIF (ng/mL)	41.9 (36.2–45.0)	52.6 (43.0–71.3)	0.0063
ESR (mm/h)	28.1 (23.4–34.9)	42.6 (32.4–51.9)	< 0.0001
CRP (mg/L)	13.4 (4.5–22.7)	19.5 (13.2–22.1)	0.14
Baseline mSASSS	8 (4–11)	16 (12–22)	< 0.0001
Follow-up mSASSS	9 (5–12)	22 (18–28)	< 0.0001
Radiograph interval (months)	24 (18–28)	24 (18–30)	0.81
Annualized mSASSS change	0.3 (0–0.6)	2.1 (1.3–3.2)	< 0.0001

Values are median (IQR) for continuous variables or n (%) for categorical variables. *BASDAI* Bath Ankylosing Spondylitis Disease Activity Index, *NSAIDs* Nonsteroidal anti-inflammatory drugs, *csDMARDs* conventional synthetic DMARDs, *TNF* Tumor necrosis factor, *MIF* Macrophage migration inhibitory factor, *ESR* Erythrocyte sedimentation rate, *CRP* C-reactive protein, *mSASSS* Modified Stoke Ankylosing Spondylitis Spine Score

### MIF and inflammatory markers by progression status

Progressors had significantly higher baseline MIF levels than non-progressors [median 52.6 ng/mL (IQR 43–71.3) vs. 41.9 ng/mL (IQR 36.2–45), *p *= 0.0063], representing a median difference of 10.7 ng/mL (95% CI 4.2–18.3). ESR was also significantly higher in progressors [median 42.6 mm/h (IQR 32.4–51.9) vs. 28.1 mm/h (IQR 23.4–34.9), *p *< 0.0001]. However, CRP did not differ significantly between groups [median 19.5 mg/L (IQR 13.2–22.1) vs. 13.4 mg/L (IQR 4.5–22.7), *p *= 0.14]. No significant differences were found in hemoglobin, platelets, total leukocyte count, lipid parameters, or HbA1c (Table 3).

### Correlations between MIF and clinical parameters

**Fig. 1:**
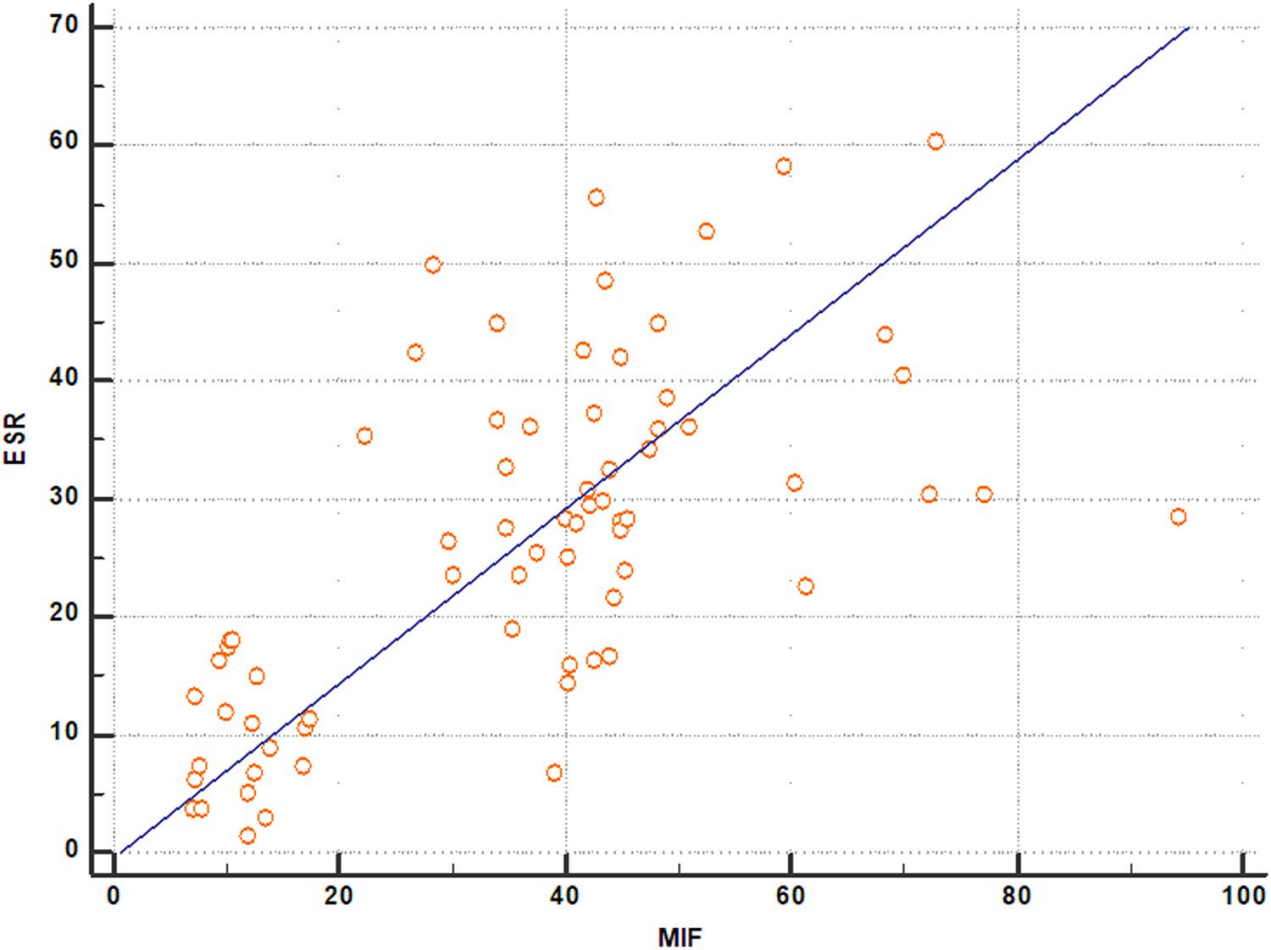
Correlation between MIF and ESR in AS patients. Scatter plot with regression line. Spearman's ρ = 0.669, *p* < 0.0001

**Fig. 2:**
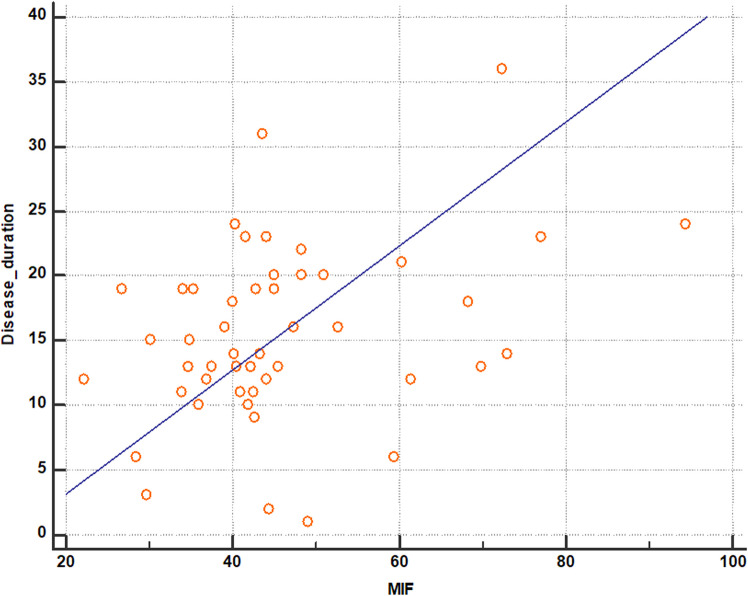
Correlation between MIF and disease duration. Scatter plot with regression line. Spearman's ρ = 0.299, *p* = 0.035

**Fig. 3:**
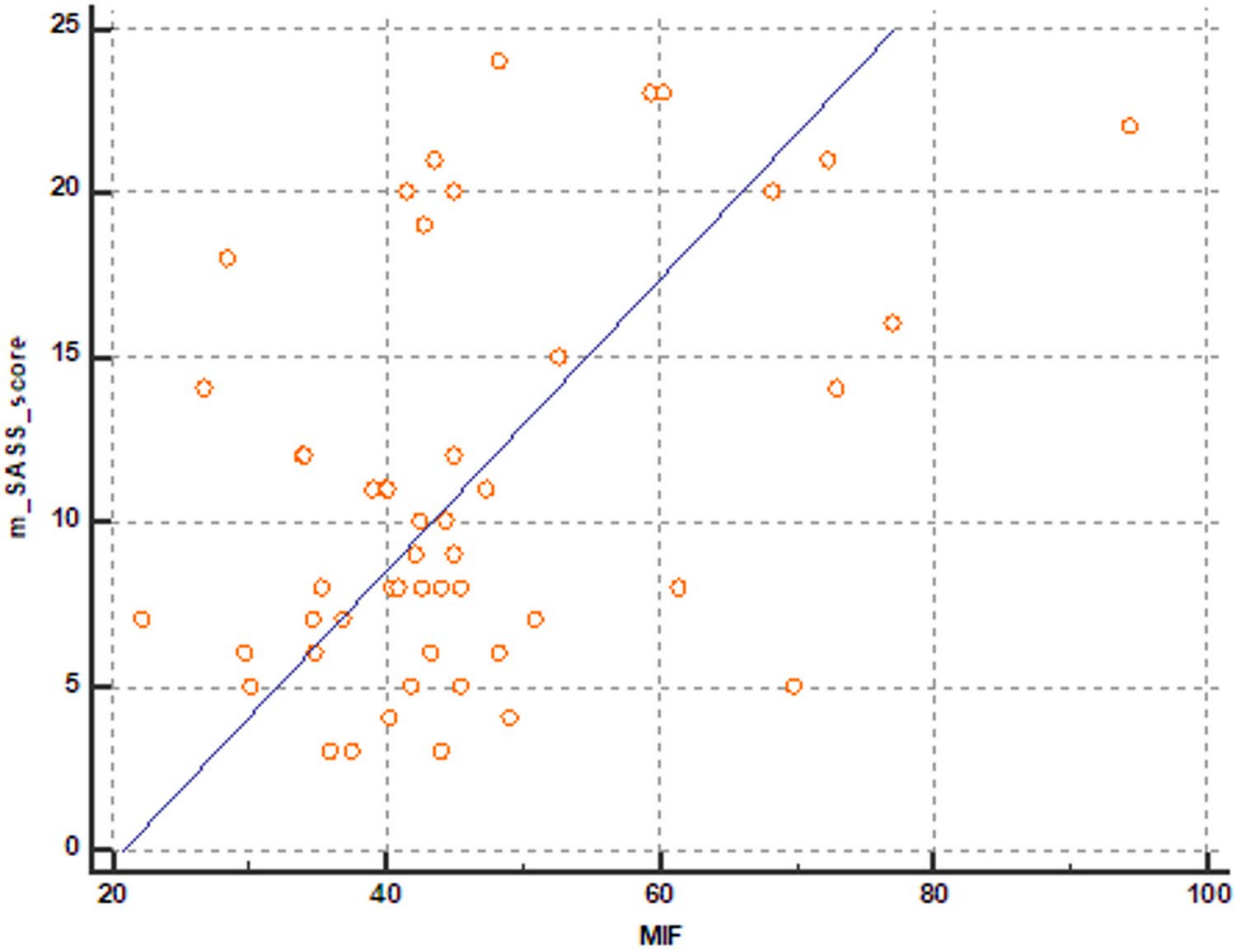
Correlation between MIF and baseline mSASSS. Scatter plot with regression line. Spearman's ρ = 0.324, *p* = 0.021

Spearman correlation analysis demonstrated significant positive correlations between baseline MIF levels and disease duration (*ρ* = 0.299, *p* = 0.035), ESR (*ρ* = 0.669, *p* < 0.0001), and baseline mSASSS (ρ = 0.324, *p* = 0.021) (Figs. 1, 2 and 3). Notably, MIF did not correlate significantly with BASDAI score (ρ = −0.008, *p* = 0.95), CRP (ρ = 0.190, *p* = 0.11), or age (ρ = 0.025, *p* = 0.84) (Table 4).

**Table 4:** Correlations between MIF and clinical parameters

Variable	Spearman's ρ	95% CI	*P* value
Age (years)	0.025	−0.26 to 0.31	0.84
Disease duration (years)	0.299	0.03 to 0.53	0.035
BASDAI score	−0.008	−0.29 to 0.28	0.95
Baseline mSASSS	0.324	0.05 to 0.55	0.021
ESR (mm/h)	0.669	0.48 to 0.80	< 0.0001
CRP (mg/L)	0.190	−0.09 to 0.44	0.11
Hemoglobin (g/dL)	−0.090	−0.36 to 0.19	0.46

*MIF* Macrophage migration inhibitory factor, *BASDAI* Bath Ankylosing Spondylitis Disease Activity Index, *mSASSS* Modified Stoke Ankylosing Spondylitis Spine Score, *ESR* Erythrocyte sedimentation rate, *CRP* C-reactive protein, *CI* Confidence interval, ρ, Spearman's rank correlation coefficient

### Independent predictors of radiographic progression

In multivariable logistic regression adjusting for age, sex, disease duration, baseline mSASSS, smoking status, and ESR, elevated baseline MIF remained independently associated with radiographic progression. For each 10 ng/mL increase in MIF, the odds of progression increased by 85% (OR 1.85, 95% CI 1.08–3.18, *p* = 0.028). Other independent predictors were current smoking (OR 5.44, 95% CI 1.18–25.1, *p* = 0.030) and ESR, where each 10 mm/h increase was associated with 73% higher odds of progression (OR 1.73, 95% CI 1.07–2.80, *p *= 0.021). Baseline mSASSS showed a trend toward association but did not reach statistical significance (OR 1.09 per unit increase, 95% CI 0.99–1.20, *p* = 0.078). Age, sex, and disease duration were not significantly associated with progression after adjustment (Table 5).

**Table 5:** Multivariable logistic regression for radiographic progression

Predictor	Odds ratio	95% CI	*P* value
Age (per 5-year increase)	1.24	0.87–1.77	0.23
Male sex	2.85	0.52–15.6	0.23
Disease duration (per 5-year increase)	1.18	0.71–1.97	0.52
Baseline mSASSS (per unit)	1.09	0.99–1.20	0.078
Current smoking	5.44	1.18–25.1	0.030
ESR (per 10 mm/h increase)	1.73	1.07–2.80	0.021
MIF (per 10 ng/mL increase)	1.85	1.08–3.18	0.028

Model fit: Hosmer–Lemeshow χ^2^ = 8.2, *p* = 0.41; all VIF < 2.5. *mSASSS* modified Stoke Ankylosing Spondylitis Spine Score, *ESR* Erythrocyte sedimentation rate, *MIF* Macrophage migration inhibitory factor, *CI* Confidence interval, *VIF* Variance inflation factor. ORs scaled to clinically interpretable units

Model diagnostics revealed no multicollinearity (all VIF < 2.5), adequate fit (Hosmer–Lemeshow χ^2^ = 8.2, *p* = 0.41), and no influential outliers (all Cook's distance < 1). Sensitivity analysis using Firth's penalized regression yielded nearly identical results (MIF OR 1.82 per 10 ng/mL, 95% CI 1.06–3.14, *p *= 0.031; ESR OR 1.71 per 10 mm/h, 95% CI 1.05–2.77, *p *= 0.032).

### Discriminative performance of MIF for predicting progression

**Fig. 4:**
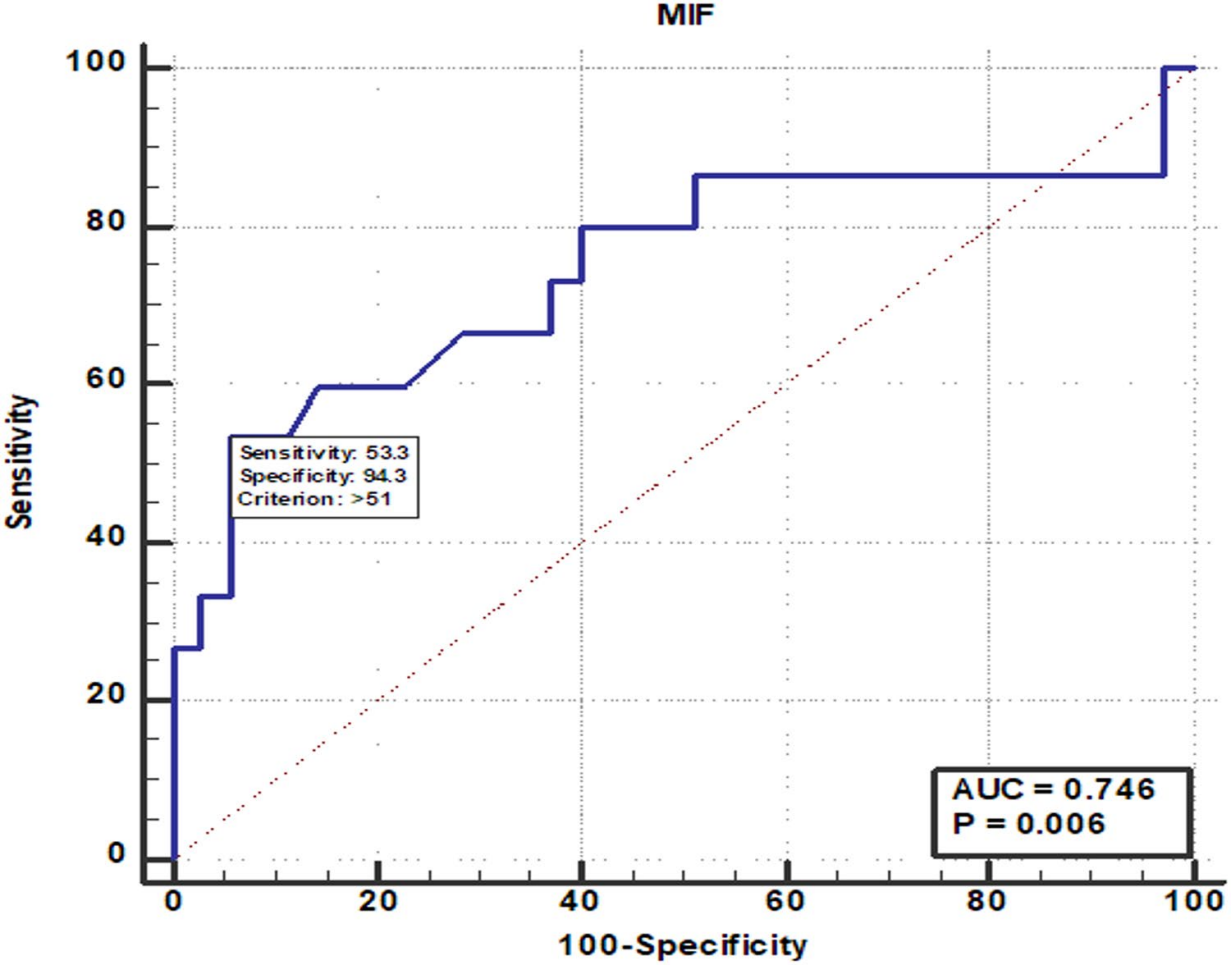
ROC curve of MIF for predicting progression. AUC = 0.746 (95% CI 0.599–0.893), *p* = 0.0056. Optimal cut-off > 51 ng/mL indicated

ROC curve analysis demonstrated that baseline MIF discriminated progressors from non-progressors with AUC of 0.746 (95% CI 0.599–0.893, *p* = 0.0056) (Table 6, Fig. 4). Using Youden's index, the optimal cut-off was MIF > 51 ng/mL, which yielded: sensitivity 53.3% (95% CI 26.6–78.7%), specificity 94.3% (95% CI 80.8–99.3%), positive predictive value (PPV) 80.0% (95% CI 44.4–97.5%), and negative predictive value (NPV) 82.5% (95% CI 67.2–92.7%). These PPV and NPV values apply to the observed 30% prevalence of progressors in this cohort; in populations with different progression rates, these predictive values would differ accordingly.

**Table 6:** ROC analysis of MIF for predicting progression

Performance metric	Value	95% CI
AUC	0.746	0.599–0.893
P value	0.0056	—
Optimal cut-off	> 51 ng/mL	—
Sensitivity	53.3%	26.6–78.7%
Specificity	94.3%	80.8–99.3%
Positive predictive value	80.0%	44.4–97.5%
Negative predictive value	82.5%	67.2–92.7%

*MIF* macrophage migration inhibitory factor, *AUC* area under ROC curve, *ROC* receiver operating characteristic, *CI* confidence interval. PPV/NPV calculated at 30% prevalence of progressors observed in this cohort

To assess incremental value, we compared the discriminative performance of models including: (1) clinical factors only (age, sex, disease duration, baseline mSASSS, smoking), (2) clinical factors + ESR, and (3) clinical factors + ESR + MIF. Adding MIF to the model with clinical factors and ESR numerically improved discrimination (ΔAUC = 0.047, *p *= 0.18), though this did not reach statistical significance in this small sample. At the population level, using MIF > 51 ng/mL as a screening threshold would correctly identify 8 of 15 progressors (sensitivity 53%) while correctly excluding 33 of 35 non-progressors (specificity 94%), with only 2 false positives.

In secondary analyses, ESR alone had AUC 0.802 (95% CI 0.677–0.927), slightly higher than MIF alone. However, the combination of MIF and ESR (both as continuous variables) achieved the highest discrimination (AUC 0.831, 95% CI 0.717–0.945), suggesting complementary prognostic information.

## Discussion

This retrospective cohort study demonstrates that baseline serum MIF levels are independently associated with subsequent radiographic progression in AS patients, even after adjustment for key confounding factors including age, sex, disease duration, baseline radiographic damage, smoking, and ESR. While the sensitivity of elevated MIF for identifying progressors is modest (53%), the high specificity (94%) suggests that MIF > 51 ng/mL may be useful for identifying patients at higher risk of progressive spinal damage. These findings support the hypothesis that MIF plays a dual role in AS pathogenesis by contributing to both inflammation and pathological new bone formation [9].

### MIF elevation in AS and correlation with inflammatory markers

Our finding of markedly elevated MIF in AS patients compared to healthy controls (median 43.0 vs. 11.2 ng/mL, representing a nearly fourfold difference) is consistent with prior reports. Karakoc et al. reported MIF elevation in AS (12.2 ± 7.7 ng/mL vs. 7.5 ± 3.7 ng/mL in controls) [10], though with lower absolute values, likely reflecting methodological differences in ELISA protocols, sample handling, or patient populations. The moderate positive correlation between MIF and ESR (ρ = 0.669) observed in our cohort aligns with MIF's established role as an upstream regulator of inflammatory cascades and sustainer of pro-inflammatory cytokine production [8].

Importantly, healthy controls were clinically screened to exclude rheumatic, autoimmune, and overt inflammatory conditions. Accordingly, the observed elevation in serum MIF in patients with AS is more likely to reflect disease-related pathophysiology rather than nonspecific inflammatory background.

### MIF as an independent predictor of progression

A key finding is that baseline MIF remained independently associated with progression after adjusting for multiple confounders that differed between progressors and non-progressors (age, sex, disease duration, baseline mSASSS, smoking, ESR). The adjusted OR of 1.85 per 10 ng/mL increase indicates that a patient with MIF of 60 ng/mL has approximately 3.4-fold higher odds of progression compared to a patient with MIF of 40 ng/mL, all else being equal. This independent association supports MIF's role as a pathogenic mediator rather than merely a marker of general inflammation.

Our findings align with and extend the seminal work of Ranganathan et al., who demonstrated in a prospective Australian cohort that baseline MIF independently predicted mSASSS progression over 2 years [9]. Importantly, their mechanistic studies revealed that MIF directly stimulates osteoblast differentiation and mineralization through Wnt/β-catenin pathway activation, providing biological plausibility for MIF's prognostic value. Our study validates these findings in an independent Middle Eastern cohort with retrospective outcome assessment, though prospective replication remains important.

The independent association of smoking with progression in our multivariable model (OR 5.44) is consistent with extensive literature documenting smoking as a risk factor for AS progression [18]. The mechanisms likely involve multiple pathways including promotion of systemic inflammation, direct effects on cartilage metabolism, and potential interactions with immunoregulatory pathways [19]. Whether MIF and smoking-related mechanisms interact synergistically warrants investigation.

### Clinical utility and interpretation of the MIF threshold

The ROC-derived threshold of MIF > 51 ng/mL demonstrated high specificity (94%) but modest sensitivity (53%). From a clinical decision-making perspective, this profile suggests MIF may be most useful as a "rule-in" biomarker: patients with MIF > 51 ng/mL have a high probability of progression (PPV 80%) and should be prioritized for strict radiographic monitoring and potential treatment intensification (e.g., early initiation of biologic therapy, more frequent monitoring). Conversely, the modest sensitivity means that normal MIF levels do not exclude progression risk—other clinical factors must be considered.

The NPV of 82.5% indicates that among patients with MIF ≤ 51 ng/mL, approximately 18% will still progress, likely driven by other pathogenic mechanisms not captured by MIF levels. This underscores that radiographic progression in AS is multifactorial, involving genetic factors (beyond HLA-B27), biomechanical stress, other inflammatory mediators (e.g., IL-17, IL-23), and individual variation in bone metabolism.

Importantly, the added value of MIF over ESR alone was numerically positive but not statistically significant in this sample (ΔAUC = 0.047, *p *= 0.18). The lack of statistical significance regarding the incremental value may reflect limited power due to the small sample size (*n* = 15 progressors). However, the stability of the association in our sensitivity analysis using Firth’s penalized regression suggests that the signal is robust despite the limited event rate. Larger studies are needed to definitively establish whether MIF provides clinically meaningful incremental value beyond conventional inflammatory markers. However, the strong correlation between MIF and ESR (ρ = 0.669) combined with MIF's independent association in multivariable analysis suggests these markers may capture partially overlapping but distinct aspects of AS pathophysiology.

### Pathogenic mechanisms linking MIF to progression

The biological basis for MIF's dual role in AS is increasingly understood. MIF not only sustains production of pro-inflammatory cytokines (TNF-α, IL-1β, IL-6, IL-17) that drive entheseal and synovial inflammation [8], but also directly influences bone metabolism. Ranganathan et al. showed that MIF stimulates osteoblast differentiation in a dose-dependent manner and upregulates genes involved in osteogenesis [9]. Furthermore, MIF triggers stabilization of β-catenin, a key mediator of the Wnt signaling pathway critical for bone formation.

Additionally, Nakamura et al. recently demonstrated that neutrophil-derived MIF exacerbates spondyloarthritis in mouse models by inducing pathogenic TH17 phenotypes in regulatory T cells [20], connecting MIF to the IL-23/IL-17 axis that is central to AS pathogenesis. This provides a mechanistic link between MIF and the inflammatory milieu that promotes both active inflammation and subsequent reparative/osteoproliferative processes.

The finding that MIF did not correlate with BASDAI score (ρ = −0.008, *p* = 0.95) is intriguing and suggests that MIF may reflect structural disease activity independent of symptomatic disease activity. This dissociation has important clinical implications, as it suggests MIF might identify patients at risk for "silent" radiographic progression despite controlled symptoms—a well-recognized phenomenon in AS [21].

### Therapeutic implications

Current AS therapies targeting TNF-α and IL-17 effectively control inflammation and improve symptoms, but their effects on long-term radiographic progression remain controversial [22, 23]. Some observational data suggest that early and sustained TNF inhibition may slow progression [24], but clinical trial and extension-study data have shown variable radiographic results [25, 26]. Given that MIF acts upstream of TNF-α and IL-17 and directly influences osteoblast activity, MIF-targeted therapies might offer advantages in preventing structural damage.

Small-molecule MIF inhibitors and anti-MIF monoclonal antibodies are in early-stage development for various inflammatory conditions [8]. Our findings, combined with Ranganathan et al.'s mechanistic data [9], support the potential utility of MIF inhibition in AS, particularly for high-risk patients identified by elevated MIF levels. Additionally, interventions that modulate endogenous MIF regulation—such as microRNA-based therapies targeting miR-451 [27]—represent novel therapeutic avenues warranting exploration.

### Strengths and limitations

Strengths of this study include: (1) rigorous radiographic assessment with blinded independent dual readers achieving high inter-reader reliability (ICC = 0.94), (2) comprehensive multivariable adjustment for key confounders including baseline radiographic damage, (3) detailed MIF assay methodology with batch analysis minimizing inter-assay variability, and (4) strict clinical screening of the control group to minimize the likelihood of overt inflammatory or autoimmune conditions.

However, important limitations must be acknowledged. First, the retrospective cohort design limits causal inference; while temporal sequence is preserved (MIF measured before progression ascertainment), unmeasured confounding remains possible. Prospective studies with serial MIF measurements are needed to establish causality and determine whether MIF changes precede progression. Second, the relatively small sample size, particularly the 15 progressors, limits statistical power for subgroup analyses and multivariable modeling. Our multivariable model included 7 predictors with 15 outcome events (2.1 events per variable), below traditional guidelines [17], necessitating cautious interpretation. However, sensitivity analyses using penalized regression yielded consistent results, increasing confidence.

Third, we used a pragmatic 1 unit/year mSASSS change threshold to define progression. While this threshold is clinically meaningful and used in prior studies [9, 15], it is somewhat arbitrary—some experts suggest 2 units/year represents more definitive progression [28]. Analyses using alternative thresholds would strengthen findings. Fourth, we relied on a single baseline MIF measurement; serial measurements were not available to determine if MIF levels fluctuate with disease activity or treatment response over the follow-up period. Fifth, we did not measure synovial fluid or tissue MIF levels, which might better reflect local disease activity at sites of pathology. Sixth, we did not assess CD74 (the MIF receptor) expression or MIF gene polymorphisms (particularly the −173 G/C promoter variant [29]), which could influence MIF activity and disease susceptibility.

Seventh, the radiographic interval varied (median 24 months, range 12–36), though this did not differ between progressors and non-progressors and was accounted for by annualizing change scores. Eighth, medication use (particularly TNF inhibitors) might influence both MIF levels and progression, though only 14% of our cohort used TNF inhibitors and this did not differ between groups. Finally, generalizability to other populations requires validation, as our cohort was recruited from tertiary referral centers in Egypt and may not represent community-based AS patients or other ethnic groups.

### Future directions

Future research should address: (1) prospective validation of MIF's prognostic value in larger, multi-center cohorts with standardized follow-up intervals, (2) serial MIF measurements to determine whether changes in MIF levels correlate with changes in progression rate and whether MIF responds to biologic therapies, (3) assessment of MIF's prognostic value in early/non-radiographic axial spondyloarthritis before structural damage occurs, (4) integration of MIF with other emerging biomarkers (e.g., matrix metalloproteinases, bone turnover markers) into multivariable prognostic models, (5) cost-effectiveness analyses of MIF-based risk stratification strategies, and (6) investigation of MIF as a therapeutic target in AS, including clinical trials of MIF inhibitors.

## Conclusion

Baseline serum MIF is independently associated with subsequent radiographic progression in AS patients after adjustment for age, sex, disease duration, baseline radiographic damage, smoking, and ESR. The high specificity of elevated MIF (> 51 ng/mL) for identifying progressors suggests potential utility for risk stratification, though the modest sensitivity indicates that MIF should be interpreted in conjunction with other clinical factors. These findings support MIF's role as both a pathogenic mediator linking inflammation to new bone formation and a candidate prognostic biomarker in AS. Given the modest sample size and exploratory nature of our multivariable modeling, prospective validation in larger cohorts is essential before MIF testing can be recommended for routine clinical use. Investigation of MIF as a therapeutic target also warrants consideration.

## Data Availability

The datasets generated and analyzed during the current study are available from the corresponding author upon reasonable request and with appropriate ethical approval for data sharing.
